# Effects of Diets With Different Protein Levels on Lipid Metabolism and Gut Microbes in the Host of Different Genders

**DOI:** 10.3389/fnut.2022.940217

**Published:** 2022-06-15

**Authors:** Kaijun Wang, Xiaomin Peng, Anqi Yang, Yiqin Huang, Yuxiao Tan, Yajing Qian, Feifei Lv, Hongbin Si

**Affiliations:** ^1^State Key Laboratory for Conservation and Utilization of Subtropical Agro-bioresources, College of Animal Science and Technology, Guangxi University, Nanning, China; ^2^Animal Nutritional Genome and Germplasm Innovation Research Center, College of Animal Science and Technology, Hunan Agricultural University, Changsha, China

**Keywords:** protein, lipid, gut, bacterial community, microbiota

## Abstract

The purpose of this experiment was to investigate the effects of different protein levels on lipid metabolism and gut microbes in mice of different genders. A total of 60 mice (30 female and 30 male) were randomly assigned to six groups and fed female mice with low protein diet (FLP), basal protein diet (FBD), and high protein diet (FHP). Similarly, the male mice fed with low protein diet (MLP), basal protein diet (MBD), and high protein diet (MHP). The low protein diet contained 14% CP, the basal diet contained 20% CP, and the high protein diet contained 26% CP. The results of the study showed that both basal and high protein diets significantly reduced the perirenal adipose tissues (PEAT) index in male mice compared to low protein diet (*p* < 0.05). For the gut, the FHP significantly increased the relative gut weight compared to the FBD and FLP (*p* < 0.05). At the same time, the FHP also significantly increased the relative gut length compared with the FBD and FLP (*p* < 0.05). The MHP significantly increased TC concentration compared with the MLP (*p* < 0.05), and the MBD tended to increase TC concentration compared with the MLP in serum (*p* = 0.084). The histomorphology result of the jejunum and ileum showed that a low protein diet was beneficial to the digestion and absorption of nutrients in the small intestine of mice. While different protein levels had no effect on the total number of fecal microbial species in mice, different protein levels had a significant effect on certain fecal microbes in mice, the absolute abundance of *Verrucomicrobia* in the feces of male mice was significantly higher in both high and basal protein diets than in the low protein diet (*p* < 0.05). The high protein diet significantly reduced the absolute abundance of *Patescibacteria* in the feces of female mice compared to both the basal and low protein diets (*p* < 0.05). The absolute abundance of *Patescibacteria* in male feces was not affected by dietary protein levels (*p* > 0.05). Taken together, our results suggest that a low protein diet can alter fat deposition and lipid metabolism in mice, and that it benefited small intestinal epithelial structure and microbes.

## Introduction

Nutrients are essential elements for maintaining health in all living organisms. Various nutrient regulatory systems exist between different organisms in order to correctly sense the nutrient environment in which they live (1). Normally, when a shortage/excess of each nutrient is detected in body tissues, some specific signaling pathways are activated, and then responsive metabolic responses are triggered (1). Protein is one of the most important nutrients, as nearly half of the dry weight of the mammalian body is made up of proteins with an incredible variety of biological functions (2). Obesity has become a major public health problem in China. In the most recent national survey, more than half of Chinese adults were overweight or obese, according to criteria for the Chinese population (3). Overweight and obesity were responsible for 11.1% of non-communicable disease (NCDs) related deaths in 2019, an increase of 5.7% from 1990 (4). These circumstances also lead to substantial health expenditures by countries to manage NCDs (5). While overweight and obesity are rapidly increasing, China has also undergone social, economic and environmental transformations. Lifestyle factors such as eating habits and physical activity have changed dramatically (6, 7). Weight loss is a major goal of reducing the risk of diabetes in humans (8). One study suggested a low-fat diet and energy restriction for obesity prevention and weight loss, but a high-protein diet is a popular alternative (9). Traditional weight loss strategies typically advise overweight and obese individuals to reduce fat intake to reduce energy intake by 500–750 calories (10). However, a high protein diet can firstly increase the body’s satiety and also increase energy expenditure, so it has become an alternative to energy restriction for weight loss (11–13).

It should be noted that the long-term effects of high protein diet, especially when combined with high fat diet, remain controversial (14). The results of systematic reviews and meta-analyses suggest that the long-term efficiency of high protein diet to induce weight loss in humans is uncertain unless dietary intervention is combined with energy restriction (15–17). While human trials have examined the ability of the high protein diet to induce weight loss in obese patients, the effects of the high protein diet in rodents were primarily conducted in preventing the development of obesity. In order to study the complex relationship between host and microbes in general, it is crucial to better understand the interactions between host and gut microbes. This can be achieved by measuring the molecules that contribute to this interaction, especially the metabolites formed by the microbiota that are available for uptake by the host. Gut microbes play key roles in animal health, including digestion of food, metabolism, regulation of immunity, and defense against invading pathogens (18–20). Many animal experiments have reported that high fat/high protein diet can effectively reduce or even prevent the development of obesity (21–26), but the ability of high protein diet to reverse obesity in rodents and its effects on fat metabolism and changes in gut microbes are far from clear. The current global obesity epidemic requires effective weight loss strategies in addition to effective methods to prevent weight gain.

The low protein diet is critical for addressing environmental concerns and conserving protein resources, but their impact on the gut microenvironment is not fully understood. Given the popularity of high protein diet, we here aimed to investigate whether diets with different protein levels can affect lipid metabolism in mice. Furthermore, we explored whether the improvement of lipid metabolism at different protein levels also affected gut microbes. Using mice as a model, it is possible to systematically study the importance of different protein levels on fat metabolism in mice, and to evaluate the possible harmful or beneficial effects of diets with different protein levels on intestinal tissue structure.

## Materials and Methods

### Animals and Dietary Treatments

The studies were approved by the Laboratory Animal Welfare and Animal Experimental Ethical Inspection Committee at the Guangxi University (Nanning, China).

Four-week-old male C57 mice (specific pathogen-free) were purchased from SLAC Laboratory Animal Central (Changsha, China). After a 1-week adaptation period, the mice were housed in a controlled environment (temperature: 25 ± 2°C, relative humidity: 45–60%, and a 12-h light–dark cycle), with free access to food and drinking water during the experiment. The diet of mice was mainly composed of corn, soybean meal, beer yeast, casein and lard. The low protein diet contained 14% CP, the basal diet contained 20% CP, and the high protein diet contained 26% CP. A total of 30 male mice of similar weight were randomly grouped into the low protein diet (MLP), basic diet (MBD), and high protein diet (MHP). Similarly, 30 female mice of similar weight were randomly grouped into the low protein diet (FLP), basic diet (FBD), and high protein diet (FHP). Throughout the experiment, the body weight of mice was measured weekly for 4 weeks. Fecal samples were collected and stored at –80°C until further analysis. At the end of the experiment, all mice were fasted overnight and killed by cervical dislocation with sodium pentobarbital anesthesia, and all efforts were made to minimize suffering. After killing, blood, subcutaneous adipose tissues (SAT), abdominal adipose tissues (AAT), perirenal adipose tissues (PEAT), liver, jejunum, ileum, and fecal contents were collected for further analyses.

### Analysis of Biochemical Parameters in Blood Samples

Serum samples were separated after centrifugation at 3,000 rpm for 10 min at 4°C. An automatic biochemistry analyzer was used to test serum biochemical parameters (27), including total cholesterol (TC, 2021061K), high density lipoprotein (HDL, 2021061K), low density lipoprotein (LDL, 2021061K) and triglycerides (TG, 2021051K).

### Histology Analysis

The jejunal and ileal tissues were removed and fixed in 4% formaldehyde solution, after which the fixed tissues were paraffin-embedded and the jejunal and ileal tissues blocks were cut into 5 μm sections, and stained with hematoxylin and eosin. In the present study, we used the pre-defined method which was reported to define the lesion (28). In each section, villus height (VH) and crypt depth (CD) were measured using a light microscope with a computer-assisted morphometric system. VH was defined as the distance from the villus tip to the crypt mouth, and CD from the crypt mouth to the base.

### Microbiota Analysis

Total genome DNA from fecal samples was extracted for amplification using a specific primer with a barcode (16S V3–V4). Sequencing libraries were generated and analyzed according to our previous study (29).

### Statistical Analyses

Statistical analyses between the means of each group were analyzed by using one-way ANOVA (one-way analysis of variance) followed by Duncan comparison range tests through SPSS 22.0. Statistical significance was set at *p* < 0.05.

## Results

### Body, Adipose Tissue, and Gut Weight or Length

**FIGURE 1:**
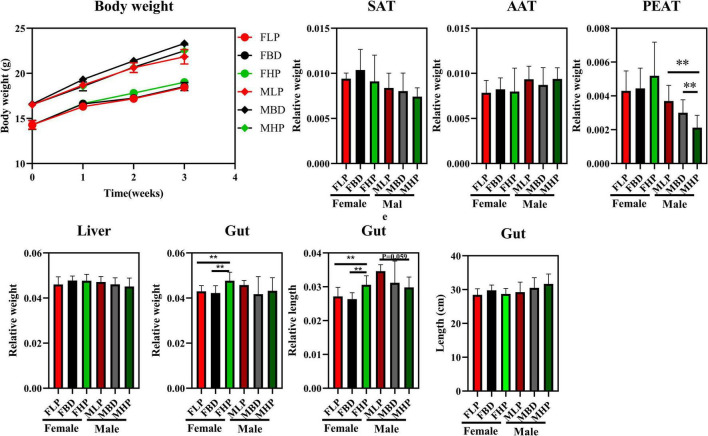
Effects of diets with different protein levels on body weight, lipid accumulation, liver weight and gut appearance in mice.

As shown in Figure 1, the three different ratios of protein had no significant effect on the final body weight of male mice (*p* > 0.05). At the same time, the different levels of protein had no significant effect on body weight of female mice (*p* > 0.05). Although different ratios of protein had no significant effect on SAT and AAT indexes in female and male mice, both MBD and MHP significantly reduced the PEAT index compared to MLP (*p* < 0.05). There was no significant effect on liver index of female and male mice by protein ratios (*p* > 0.05). For the gut, the high protein diet significantly increased the relative gut weight of female mice compared to the basal and low protein diets (*p* < 0.05). At the same time, the high protein diet also significantly increased the relative gut length of female mice compared with the basal and low protein diets (*p* < 0.05). However, different levels of protein concentration diet had no significant effect on the relative weight and relative length of gut to male mice. Furthermore, there was no significant difference in gut length between female and male mice between diets with different protein levels (*p* > 0.05).

### Lipid Metabolism Index in Serum

**FIGURE 2:**
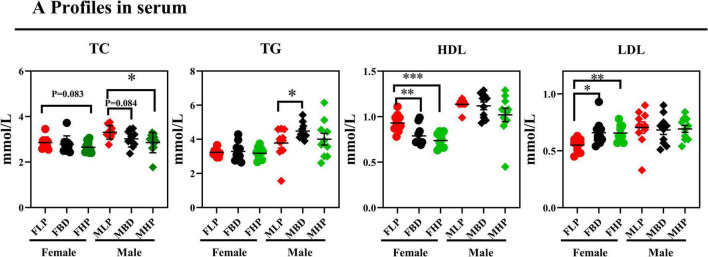
Effects of diets with different protein levels on serum lipid levels in mice.

As shown in Figure 2, the FHP tended to increase serum TC concentration compared with the FLP (*p* = 0.083). However, the MHP significantly increased TC concentration compared with the MLP (*p* < 0.05), and the MBD tended to increase TC concentration compared with the MLP in serum (*p* = 0.084). Although diets with different protein levels in female mice had no significant effect on serum TG concentrations (*p* > 0.05), however, the MBD significantly increased the content of TG in serum compared with the MLP (*p* < 0.05). The FLP resulted in significantly higher serum HDL than the FBD and FHP (*p* < 0.05), whereas both the FHP and FBD significantly increased serum LDL level in female mice (*p* < 0.05). Different levels of protein diets did not significantly alter serum HDL and LDL levels in male mice (*p* > 0.05).

### Histomorphological Analysis of Small Intestine

**FIGURE 3:**
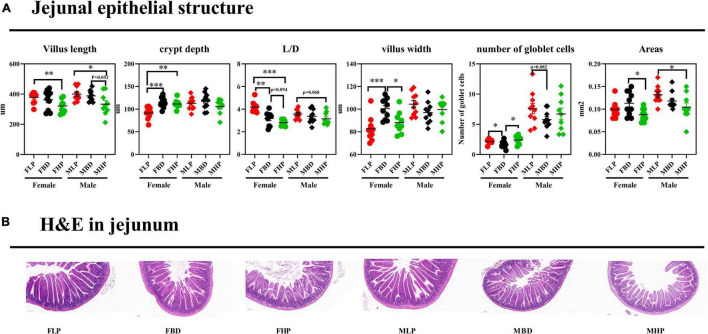
Effects of diets with different protein levels on the morphology of jejunal epithelial tissue in mice. **(A)** The structure of the jejunal epithelial tissue. **(B)** Light microscopy cross-section of jejunal tissue.

The jejunal tissue morphology under three different dietary treatments to female and male mice was shown in Figure 3. According to our study, jejunal villus length decreased with increasing dietary protein levels in both male and female mice, and a high protein diet resulted in a significantly lower jejunal villus length than a low protein diet (*p* < 0.05). In female mice, both the basal diet and the high protein diet significantly increased CD in the jejunum (*p* < 0.05), while the three dietary treatments had no effect on CD in male mice (*p* > 0.05). With the increase of dietary protein level, the ratio of jejunal villus length to CD (L/D) decreased in female mice, and the L/D ratio of basal diet and high protein diet was significantly lower than that of low protein diet (*p* < 0.05). The results for male mice showed that increased protein levels led to a decrease in jejunal L/D, but the difference was not significant (*p* > 0.05). The jejunal villus width of the FLP was significantly lower than that of the FBD (*p* < 0.001) and the FBD was significantly higher than that of the FHP (*p* < 0.05), and the protein concentration had no effect on the villus width of the male mice (*p* > 0.05). The number of jejunal goblet cells on the FBD was significantly lower than that on the FLP and FHP (*p* < 0.05), and the number of jejunal goblet cells in male mice also tended to be higher on the low protein diet than on the basal diet (*p* = 0.082). The high protein diet resulted in significantly lower jejunal villus area in female mice than on the basal diet (*p* < 0.05), while the high protein diet in male mice resulted in significantly lower jejunal villus area than the low protein diet (*p* < 0.05).

**FIGURE 4:**
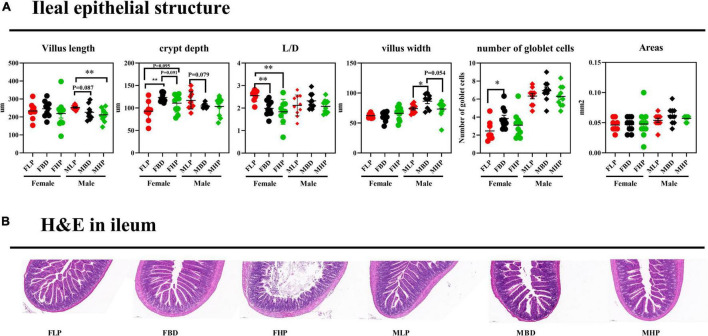
Effects of diets with different protein levels on the morphology of ileal epithelial tissue in mice. **(A)** The structure of the ileal epithelial tissue. **(B)** Light microscopy cross-section of ileal tissue.

The ileal tissue morphology under three different dietary treatments to female and male mice was shown in Figure 4. In this study, the ileal villus length significantly decreased in high protein diet than low protein diet for male mice (*p* < 0.05), and three protein diets had no significantly difference on villus length of female mice (*p* > 0.05). In female mice, the FBD significantly increased CD in the ileum compared with the FLP (*p* < 0.05), while the MBD tended to decreased CD than MLP in the ileum of male mice (*p* = 0.079). With the increase of dietary protein level, the ratio of ileal L/D decreased in female mice, and the L/D ratio of basal diet and high protein diet was significantly lower than that of low protein diet (*p* < 0.05). The results for male mice showed that various protein level diets had no significant difference on ileal L/D (*p* > 0.05). The ileal villus width of the MBD was significantly higher than that of the MLP (*p* < 0.05), and the MBD tended to increase the ileal villus width than that of the MHP in male mice (*p* = 0.054). The protein concentration had no effect on the ileal villus width of the female mice (*p* > 0.05). The number of ileal goblet cells on the FBD was significantly higher than that on the FLP (*p* < 0.05), and the number of ileal goblet cells in male mice had no significant difference between the low and high protein diet in female mice (*p* > 0.05). Meanwhile, different levels of protein diet had no effect on the number of goblet cells in the ileum of male mice (*p* > 0.05). Finally, different levels of protein diet had no significant effect on ileal villus area in both female and male mice (*p* > 0.05).

### Fecal Bacterial α and β Diversity

**FIGURE 5:**
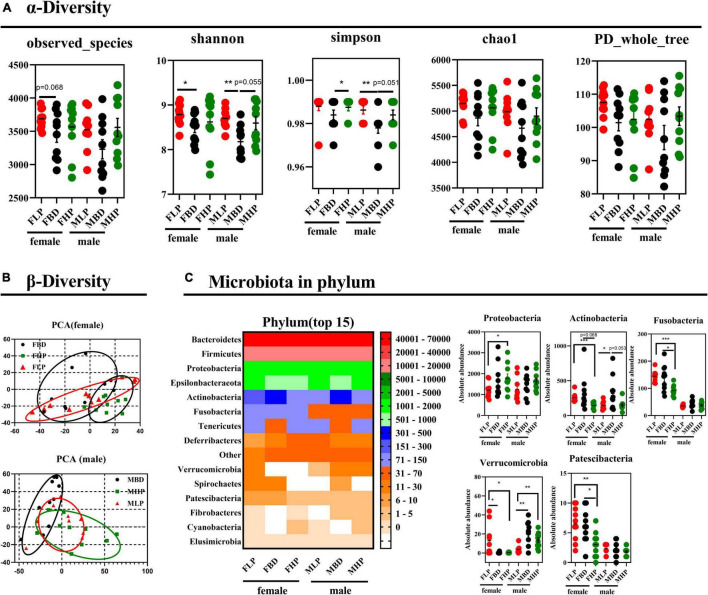
Effects of diets with different protein levels on **(A)** α diversity, **(B)** β diversity, and **(C)** phylum-level bacteria in the fecal microbiota of mice.

The fecal bacterial α diversity in male and female mice with different levels of protein diets was shown in Figure 5A. The results showed that the FBD tended to reduce observed species compared to the FLP (*p* = 0.068), while no significant difference was found between the high protein diet and the other two groups (*p* > 0.05). There were no significant differences in fecal observed species in male mice fed the diets of the three protein levels (*p* > 0.05). The low protein diet significantly increased the Shannon index compared with the basal diet in female mice (*p* < 0.05), while the high protein diet did not produce significant differences with the other two groups on Shannon index in female mice (*p* > 0.05). In the fecal bacteria of male mice, the MLP significantly increased the Shannon index of bacteria compared to the MBD (*p* < 0.05), while the MHP showed a trend to increase the Shannon index compared to the MBD (*p* = 0.055). The high protein diet significantly improved Simpson index over basal diet in female mice (*p* < 0.05), whereas high protein diets over basal diets tended to increase Simpson index in male mice (*p* = 0.051). At the same time, the low protein diet also significantly improved the Simpson index in male mice compared to the basal diet (*p* < 0.05). There were no significant differences in the fecal bacterial chao1 index and PD_whole_tree between the three diets in either female or male mice (*p* > 0.05). As shown in Figure 5B, the principal components of female mice were similar between the different protein levels and did not produce significant separation. In male mice, however, there was a distinctly different cluster of microbiomes between the basal and high protein diets.

### Diets With Different Protein Levels Affect Fecal Microbiota Composition

The bacterial effects of different protein levels on the top 15 bacteria at the phylum level of the fecal microbiota of male and female mice were shown in Figure 5C. The high protein diet significantly increased the absolute abundance of *Proteobacteria* in the feces of female mice compared to the low protein diet (*p* < 0.05). The abundance of *Proteobacteria* did not make a difference in male feces (*p* > 0.05). In contrast, the high protein diet significantly reduced the absolute abundance of *Actinobacteria* in the feces of female mice compared to the low protein diet (*p* < 0.05). The abundance of *Actinobacteria* in the feces of female mice tended to decrease in the high protein diet compared with the basal protein diet (*p* = 0.068). Furthermore, the high protein diet significantly reduced the absolute abundance of *Fusobacteria* in the feces of female mice compared to the low and basal protein diet (*p* < 0.05). The absolute abundance of *Fusobacteria* in male mice was not affected by dietary protein levels (*p* > 0.05). In addition, both high protein and basal protein diets significantly reduced the absolute abundance of *Verrucomicrobia* in the feces of female mice compared to low protein diets (*p* < 0.05). Conversely, the absolute abundance of *Verrucomicrobia* in the feces of male mice was significantly higher in both high and basal protein diets than in the low protein diet (*p* < 0.05). The high protein diet significantly reduced the absolute abundance of *Patescibacteria* in the feces of female mice compared to both the basal and low protein diets (*p* < 0.05). The absolute abundance of *Patescibacteria* in male feces was not affected by dietary protein levels (*p* > 0.05).

**FIGURE 6:**
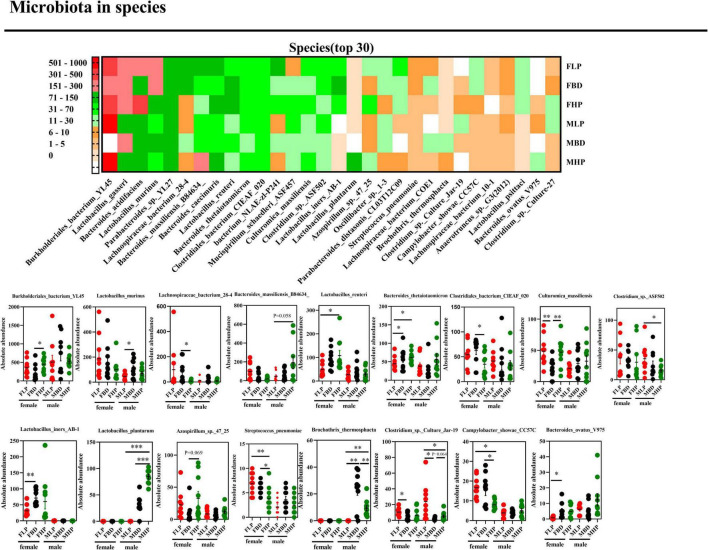
Effects of diets with different protein levels on species-level bacteria in the fecal microbiota of mice.

Downward to species level (Figure 6), we found that 17 out of 30 species were markedly changed throughout the entire stage. The high protein diet of female mice significantly increased the absolute abundances of *Burkholderiales_bacterium_*YL45 and *Culturomica_massiliensis* (*p* < 0.05), but significantly decreased the absolute abundances of *Lachnospiraceae_bacterium_*28-4, *Clostridiales_bacterium_CIEAF_*020, *Streptococcus_pneumoniae* and *Campylobacter_showae_*CC57C compared with the basal diet (*p* < 0.05). At the same time, the abundance of *Azospirillum_sp._*47_25 has an upward trend in the high protein diet compared with the basal diet in the feces of female mice (*p* = 0.069). However, the abundances of *Lactobacillus_murinus*, *Bacteroides_massiliensis_*B84634_, *Clostridium_sp._*ASF502, *Lactobacillus_plantarum* and *Brochothrix_thermosphacta* in female mice feces were not affected by protein levels (*p* > 0.05). In the fecal microbiota of male mice, a high protein diet significantly increased the absolute abundances of *Lactobacillus_plantarum* and *Brochothrix_thermosphacta* (*p* < 0.05), but significantly decreased the absolute abundances of *Clostridium_sp._*ASF502 and *Clostridium_sp._Culture_*Jar-19 compared with a low protein diet (*p* < 0.05). In addition, the high protein diet tended to increase *Clostridium_sp._Culture_*Jar-19 abundance in male mice feces compared to basal protein diet (*p* = 0.064). And in male mice fecal microbiota, the absolute abundances of *Burkholderiales_bacterium_* YL45, *Lachnospiraceae_bacterium_*28-4, *Lactobacillus_reuteri*, *Bacteroides_thetaiotaomicron*, *Clostridiales_bacterium_CIEAF_* 020, *Culturomica_massiliensis*, *Lactobacillus_iners_AB*-1, *Azospirillum_sp._*47_25, *Streptococcus_pneumoniae*, *Campy-lobacter_ showae_*CC57C, and *Bacteroides_ovatus_*V975 were not affected by dietary protein levels (*p* > 0.05).

## Discussion

In this study, female and male mice were used as experimental models to investigate the effects of different levels of protein diets on fat deposition, fecal microbiota, and small intestinal tissue structure in mice. Studies have shown that dietary protein played a role in regulating lipid biological synthesis. In some rodents’ research, genes participating in new fat production and lipid storage such as PPARγ (21), ACC (21, 30), FASN (21, 30), SCD1 (31), and SREBP1c (31, 32) were reduced by intake of HP diet. Compared with LP diet, the HP diet fat intake and lipid biological synthetic genes were lower. The HP diet reduces liver fat more effectively than the LP diet (33). For example, in mice and human research, the HP diet has proven to increase energy consumption, reduce blood glucose levels, promote fat oxidation, thereby supporting weight loss, and reducing liver fat (34–37). The HP diet may inhibit liver fat production at the molecular level to reduce liver fat (21, 22, 38). The relatively lower carbohydrate content in the HP diet may also be part of the cause of new fat production and reduced intrahepatic fat. In addition, compared with fat-generating genes, in several rats’ research, dietary protein intake has not changed the expression of genes related to lipid oxidation or substrate oxidation (30, 32). Our results showed that the HP diet is significantly reduced in the perirenal fat more than the LP and the basic diet. The reason may be that the fat intake and lipid biological synthesis may be inhibited.

The biochemical indicators of the body’s blood can not only feedback the health of the host and the strength of immune function, but also reveal the biological characteristics of different hosts (39). When the level of TC in the host’s blood rises, hypertrophymia will occur. Compared with LDL, HDL level may lead to this situation (40). Some researchers have compared the influence of LP and HP diets on obesity and their related diseases. One of the main discoveries in the system summary was that the HP solution has a favorable impact on TG and HDL (16). This was consistent with the results of the HP diet in our research reduced the HDL in the female blood. The HP diet reduced TC and HDL, but LDL has increased, indicating that the lipid metabolism in the blood has changed. These data are very important for preventing hyperlipidemia and heart and liver disease (41).

Different parts of the digestive tract assume different responsibilities. The small intestine was the key place for the host to absorb nutrients. The height of villi and the depth of crypts in the small intestine were important indicators to evaluate its digestion and absorption function. The CD reflects the speed of cell formation, while shallower crypts indicate that the cell maturity was accelerated and the secretion function is enhanced. The VH and CD could fully reflect the functional state of the small intestine (42). In the present study, jejunal villus length decreased and CD increased after mice were fed the HP diet, suggesting that the HP diet may have a potential adverse effect on jejunal function. Since the small intestine surface area (represented by the tight villus packing and long villus protrusions) showed the maximum nutrient absorption allowed (43), the reduced ratio of VH to CD in the jejunum and ileum of the HP group diet indicated nutrient absorption reduce. Crypts were where ISCs were synthesized and amplifying cells were transported, and CD could also reflect the proliferation of intestinal epithelial cells. In the present study, an increase in jejunal CD with increasing protein levels in the diet showed the possibility of intestinal epithelial proliferation, whereas an increase in epithelial proliferation with increasing protein levels in the ileum occurred only in the FLP and FBD groups. The intestinal epithelial barrier in animals was initiated by the ISC niche, which develops differentiated cell types, including Paneth cells and goblet cells (44). The increase in goblet cells in the FBD group indicated the possibility of ISC proliferation in the ileum. Thus, the microbiota in the FBD group than in the FLP group may have stimulated ISCs, improved ileal barrier function, and was supported by an increase in Lactobacillus, which has been reported to stimulate intestinal epithelial cell proliferation through Nox-mediated reactive oxygen species production (45).

Dietary protein was an essential nutrient for animals and it was necessary for the physiological functions of organs. The structural integrity of the gut and the homeostasis of the gut microbiota ensure the chemical induction and digestive functions of the gut, which were prerequisites for nutrient absorption, metabolism, and deposition. The gut microbiota was considered a key factor in maintaining gut function (46, 47), it was a complex ecosystem with nearly 100 trillion microorganisms, most of which were bacteria (48). Many factors influenced gut microbiota composition and activity, including diet, environment, and age; of these, diet was the most important (49–51). Diet played a crucial role in shaping the composition of animal gut microbiota and the state of immune responses mediated by gut microbiota (52). We explored the effect of diets with different protein levels on fecal microbial diversity in mice. In the present study, it can be concluded that the fecal community diversity of mice on a low protein diet was higher than that on a basal diet and a high protein diet, but it was observed that different protein levels had no effect on the total fecal microbial population in mice. The microbiome consists of trillions of microbial cells with high inter- and intra-species variability, so it was difficult to define a healthy gut microbiome based on the species in the gut (53, 54). However, the variability of microbial functional genes and metabolites may be low (54, 55). An increased ratio of *Firmicutes* to *Bacteroides* has been reported in obese animals (56) and was associated with host energy intake (57, 58). Some researchers have shown that the composition of different microbial communities in the digestive tract of animals was different, and the diversity and density of microbial communities gradually increased from the stomach to the hindgut (59). For the ileal microbiota, the reduced *Enterobacteriaceae* within the *Proteobacteria* phylum were thought to contain many pathogenic bacteria when dietary protein decreased by 3 percentage points (60), suggesting the potential for pathogen suppression by moderate dietary protein restriction. The *Streptococcus* and *Escherichia-Shigella* can cause a variety of infections and diseases, such as scarlet fever and bacillary dysentery (61, 62). In this study, different protein level diets had no difference in *Escherichia-Shigella* of female and male mice. Research by NEIS et al. showed that when the concentration of diet protein is reduced by 3 percentage points, the number of *Streptococcus* and *Escherichia-Shigella* declined in the ileum, and the *Streptococcus* participated in the use of amino acids (63). Therefore, the decline of link bacteria such as *Streptococcus_pneumoniae* in feces of FHP diet, it can be explained as insufficient protein substrates required for fermentation, which was consistent with previous research (64, 65). The *Lactobacillus* bacteria in the intestine could ferment the carbohydrates in the diet into lactic acid and improved the intestinal environment (66, 67). The elevation of *Lactobacillus_plantarum* and *Lactobacillus_reuteri* in the high protein diet in this study fitted this situation. An increasing on *Lactobacillus_murinus* and *Lactobacillus_iners_*AB-1 in the basal diet compared to the low protein diet may be because the carbohydrates in the basal diet were higher than the low protein diet, but the two bacteria were decreased in the high protein diet. It may be that the high protein diet weakened the intestinal barrier function. Chen et al. used pigs as an experimental model to show that the abundance of *Lachnospiraceae*, which was saccharolytic and can degrade cellulose, was increased in low protein diet (68). This was consistent with our findings that the high protein diet reduced *Lachnospiraceae_bacterium_*28-4 in the feces of female mice compared to the basal diet. Therefore, according to the results of this study, a low protein diet was more beneficial to the gut microbiome.

## Conclusion

In conclusion, this study demonstrated that diets with different protein levels can affect lipid deposition and lipid metabolism in mice. At the same time, a high protein diet weakened the small intestinal barrier structure, which weakened the host’s ability to digest and absorb nutrients. Although different protein levels had no effect on the total number of fecal microbial species in mice, different protein levels had significant effects on the structure of some fecal microbiota in mice. The reduction of *Lactobacillus_murinus* and *Lactobacillus_iners*_AB-1 on the low protein diet due to higher carbohydrates in the basal diet than the low protein diet. Besides, the high protein diet reduced *Lachnospiraceae_bacterium*_28-4 in the feces of female mice compared to the basal diet. Our study provided a more comprehensive understanding of lipid metabolism, gut barrier and fecal microbial response to diets at different protein levels in female and male mice.

## Data Availability Statement

The original contributions presented in the study are included in the article/supplementary material, further inquiries can be directed to the corresponding author.

## Ethics Statement

The studies were approved by the Laboratory Animal Welfare and Animal Experimental Ethical Inspection Committee at the Guangxi University (Nanning, China).

## Author Contributions

KW and HS designed the experiment. KW conducted the experiment and wrote the manuscript. KW, XP, AY, YH, YT, YQ, and FL collected and analyzed the data. HS revised the manuscript. All authors contributed to the article and approved the submitted version.

## Conflict of Interest

The authors declare that the research was conducted in the absence of any commercial or financial relationships that could be construed as a potential conflict of interest.

## Publisher’s Note

All claims expressed in this article are solely those of the authors and do not necessarily represent those of their affiliated organizations, or those of the publisher, the editors and the reviewers. Any product that may be evaluated in this article, or claim that may be made by its manufacturer, is not guaranteed or endorsed by the publisher.
